# Somatostatin receptor expression in small cell lung cancer as a prognostic marker and a target for peptide receptor radionuclide therapy

**DOI:** 10.18632/oncotarget.7706

**Published:** 2016-02-25

**Authors:** Constantin Lapa, Heribert Hänscheid, Vanessa Wild, Theo Pelzer, Andreas Schirbel, Rudolf A. Werner, Sabine Droll, Ken Herrmann, Andreas K. Buck, Katharina Lückerath

**Affiliations:** ^1^ Department of Nuclear Medicine, University Hospital Würzburg, 97080 Würzburg, Germany; ^2^ Institute for Pathology, University of Würzburg, 97080 Würzburg, Germany; ^3^ Department of Internal Medicine, University Hospital Würzburg, 97080 Würzburg, Germany

**Keywords:** small cell lung cancer, molecular imaging, somatostatin receptor, positron emission tomography, PET

## Abstract

Despite initial responsiveness to both chemotherapy and radiotherapy, small cell lung cancer (SCLC) commonly relapses within months. Although neuroendocrine characteristics may be difficult to demonstrate in individual cases, a relevant expression of somatostatin receptors (SSTR) on the cell surface has been described. We aimed to evaluate the prognostic value of SSTR-expression in advanced SCLC. We further examined pre-requisites for successful peptide receptor radionuclide therapy (PRRT).

21 patients with extensive stage SCLC were enrolled. All patients underwent positron emission tomography/computed tomography (PET/CT) with ^68^Ga-DOTATATE to select patients for SSTR-directed therapy. PET scans were visually and semi-quantitatively assessed and compared to SSTR2a and SSTR5 expression in biopsy samples. Peak standardized uptake values (SUV_peak_) of tumors as well as tumor-to-liver ratios were correlated to progression-free (PFS) and overall survival (OS).

In 4/21 patients all SCLC lesions were PET-positive. 6/21 subjects were rated “intermediate” with the majority of lesions positive, the remaining 11/21 patients were PET-negative. PET-positivity correlated well with histologic SSTR2a, but not with SSTR5 expression. Neither PET-positivity nor SUV_peak_ were predictors of PFS or OS. In 4 patients with intensive SSTR2a-receptor expression, PRRT was performed with one partial response and one stable disease, respectively.

SSTR-expression as detected by ^68^Ga-DOTATATE-PET and/or histology is not predictive of PFS or OS in patients with advanced SCLC. However, in patients exhibiting sufficient tracer uptake, PRRT might be a treatment option given its low toxicity and the absence of effective alternatives.

## INTRODUCTION

Small cell lung cancer (SCLC) represents 15% of all lung cancers and occurs predominantly in smokers [1]

Due to its rapid doubling time, high growth fraction, and the early development of metastases, only one-third of patients are diagnosed with localized disease [2]. Although SCLC is highly responsive to both chemotherapy and radiotherapy, it commonly relapses within months despite treatment [3, 4].

SCLC represents the most aggressive member of a family of tumors with neuroendocrine features such as typical and atypical carcinoids or large cell neuroendocrine carcinomas (LCNEC). Although neuroendocrine characteristics may be difficult to demonstrate in individual cases, a relevant expression of somatostatin receptors (SSTR) on the cell surface has been described [5, 6].

The ^68^Ga/^177^Lu/^90^Y-labelled compound DOTATATE/-TOC has been shown to reliably and selectively bind to SSTR2a (and SSTR5). It is widely used in imaging (^68^Ga) and therapy (^177^Lu, ^90^Y) of neuroendocrine tumors [7, 8]. The aim of this study was to evaluate the prognostic value of SSTR-expression in patients with advanced SCLC. We further examined pre-requisites for successful peptide receptor radionuclide therapy (PRRT) in this patient group.

## RESULTS

### Patients

At the time of SSTR-PET/CT, all patients presented with extensive disease and suffered from tumor progression. Metastatic sites included lymph nodes (19/21), liver (10/21), bone (10/21), adrenals (8/21), lung (5/21), brain (4/21), pleura (4/21), pancreas (1/21) and skin (1/21). During follow-up, all patients deceased with 20/21 dying from SCLC and 1 subject from pneumonia. Median PFS was 70 days (range, 2-766 d), median OS 118 days (range, 3-907 d).

### SSTR-PET/CT-Imaging

**Figure 1:**
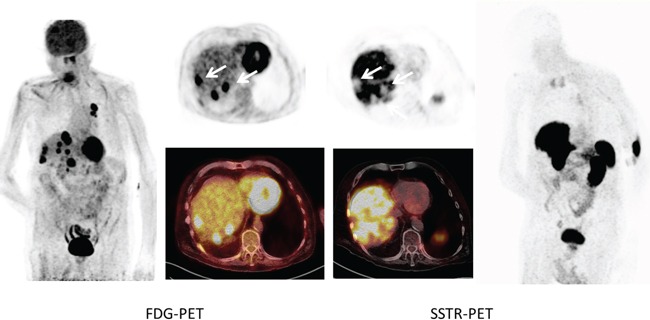
Example of SSTR-negative SCLC (patient #1) Maximum intensity projections as well as transaxial slices of FDG- and SSTR-PET/CT are displayed. The patient had multiple hypermetabolic lymph node, intrapulmonary and liver (arrows) lesions which showed intense FDG uptake. In contrast, ^68^Ga-DOTATATE PET demonstrated no relevant expression of somatostatin receptors with the liver metastases presenting as photopenic lesions (arrows).

On visual analysis, 4/21 patients were rated “positive” with all SCLC lesions displaying higher tracer uptake than liver parenchyma, 6/21 were rated “intermediate”, and 11/21 presented with ^68^Ga-DOTATATE-negative disease (Figure 1). The SUV_peak_ values and the T/L ratios of the maximum tumor to mean liver SUV are listed in Table 1. Both SUV_peak_ values (p<0.001, Kruskal-Wallis test) and T/L ratios (p<0.001, Kruskal-Wallis test) were different in the 3 groups. The highest values (SUV >20, tumor to liver ratio >2,7) were observed in the 4 patients rated positive with increased uptake in all lesions. SUV_peak_ (9.8±2.5; p=0.01, T-test) and T/L ratios (1.4±0.3; p=0.02, T-test) were lower in the intermediate group and even lower (4.6±1.5 and 0.6±0.2, respectively; p<0.001 each, T-test) in the negative group.

**Table 1:** SUV_peak_ values, T/L ratios of the peak tumor to mean liver SUV and correlation of visual PET results and immunoreactive scores (IRS) for SSTR2a and SSTR5

Patient	PET	SUVpeak	T/L	SSTR2-IRS	SSTR5-IRS
**1**	negative	6.0	0.4	0	0
**2**	positive	23.3	2.7	9	0
**3**	intermediate	9.2	1.8	1	0
**4**	negative	7.2	0.8	N/A	N/A
**5**	intermediate	10.8	1.1	0	4
**6**	positive	32.5	4.8	8	0
**7**	intermediate	14.2	1.3	0	0
**8**	intermediate	9.1	1.4	8	2
**9**	positive	35.3	4.5	12	0
**10**	positive	20.5	2.7	12	2
**11**	negative	6.2	1.0	0	0
**12**	intermediate	8.5	1.6	0	0
**13**	negative	3.6	0.6	2	0
**14**	negative	4.8	0.8	0	0
**15**	negative	3.3	0.4	0	0
**16**	negative	5.4	0.8	0	0
**17**	intermediate	7.0	0.9	0	0
**18**	negative	3.9	0.4	1	0
**19**	negative	2.5	0.4	0	0
**20**	negative	2.8	0.8	1	0
**21**	negative	4.9	0.5	N/A	N/A

For quantification of SSTR-positivity at PET, the categories “positive” (all tumor lesions PET-positive with ^68^Ga-DOTATATE uptake > liver), “intermediate” (the majority of lesions positive) and “negative” (the majority of / all tumor lesions PET-negative) are used. The IRS score is given for histologically assessed SSTR2a/5 expression.

### Correlation of ^68^Ga-DOTATATE uptake and histopathology

In 19/21 patients, imaging results could be compared to immunohistological staining for SSTR2a and SSTR5 derived from biopsies of the primary tumor (n=15) or metastases (n=4). Regarding the histological evaluation of SSTR2a expression, 1/19 samples was rated “mild” (IRS 2), 2/19 “moderate” (both IRS 8) and 3/19 “strongly” (one IRS 9, two IRS 12) positive, while 13/19 were scored negative (2/13 with IRS 1 and 11/13 with IRS 0). In contrast, 16/19 samples did not show any SSTR5 expression (all IRS 0), 1/19 was mildly (IRS 2) and 2/19 were moderately (both IRS 4) positive (Table 2).

**Table 2:** Patients' characteristics

Patient	sex	Age (y)	disease	previous Tx	Tx post PET	PFS (d)	OS (d)
**1**	m	74	LCNEC	RTx	none	2	5
**2**	f	74	SCLC	RCTx (1^st^/2^nd^ line)	PRRT (5x)	766	907
**3**	m	64	SCLC	None	CTx	161	188
**4**	f	52	SCLC	RCTx (1^st^/2^nd^ line)	CTx	69	93
**5**	f	56	SCLC	CTx (1^st^ line)	CTx	n/a	465
**6**	m	47	SCLC	RCTx (1^st^/2^nd^ line)	CTx	51	66
**7**	m	54	SCLC	RCTx (1^st^/2^nd^ line)	CTx	71	95
**8**	m	67	SCLC	surgery, RCTx (1^st^/2^nd^ line)	CTx	59	126
**9**	m	69	SCLC	CTx (1^st^/2^nd^ line)	PRRT (1x)	115	118
**10**	m	66	SCLC	CTx (1^st^/2^nd^ line)	PRRT (1x)	53	53
**11**	m	51	SCLC	RCTx (1^st^/2^nd^ line)	CTx	26	38
**12**	f	65	SCLC	RCTx (1^st^/2^nd^ line)	none	8	22
**13**	m	66	SCLC	RCTx (1^st^ line)	none	3	3
**14**	f	52	LCNEC	RCTx (1^st^ line)	CTx	308	432
**15**	m	78	LCNEC	CTx (1^st^/2^nd^ line)	CTx	83	118
**16**	f	62	SCLC	RCTx (1^st^/2^nd^ line)	CTx	324	434
**17**	m	48	SCLC	RCTx (1^st^/2^nd^ line)	PRRT (6x)	396	575
**18**	m	51	SCLC	RCTx (1^st^ line)	CTx	225	239
**19**	f	69	SCLC	RCTx (1^st^/2^nd^ line)	RTx	8	27
**20**	f	59	SCLC	RCTx (1^st^ line)	CTx	85	568
**21**	f	41	SCLC	RCTx (1^st^ line)	CTx	63	95

CTx= chemotherapy; f= female; LCNEC= large cell neuroendocrine carcinoma; m= male; OS= overall survival; PFS= progression-free survival; PRRT= peptide receptor radionuclide therapy; RCTx= radio-chemotherapy; RTx= radiotherapy; SCLC= small cell lung cancer. The age of the patients is given in years, OFS and OS in days.

**Figure 2:**
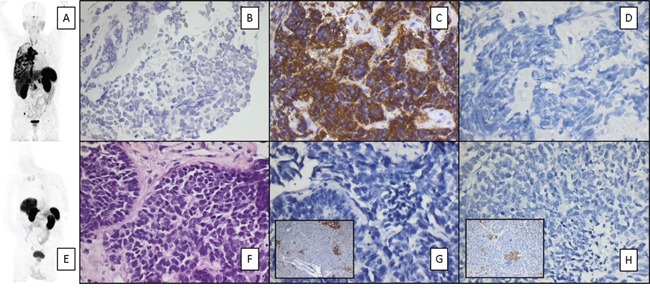
Example of SSTR-directed PET imaging matching immunohistochemical quantification of receptor expression Given are maximum intensity projections **A, E.** and immunohistochemical stainings of SCLC patients with high (Patient # 9, upper row; **C**) and low (patient #1, lower row; **G**) SSTR 2a expression. SSTR5 expression was low in both samples **D, H.** Positive controls for both SSTR2a and SSTR5 are shown in the inserts in panels **G** and **H.** Counterstaining was performed with hematoxylin **B, F.** Magnification: 200x **B, F.** and 400x **C-H.**

SUV_peak_ as well as the T/L ratios were significantly correlated to the IRS score of SSTR2a expression (Spearman's Rho: ρ=0.49 with p=0.03 and ρ=0.61 with p<0.01, respectively) but not to SSTR5 expression (Spearman's Rho: ρ=0.31 with p=0.19 and ρ=0.27 with p=0.27, respectively) (Figure 2). In the Kruskal-Wallis test for differences in the groups of PET positivity, no significant differences were found for the SSTR5 IRS score (p=0.2). A difference for the SSTR2a IRS score (p<0.01) was attributable to a significantly higher score in the 4 patients rated positive with increased uptake in all lesions; no difference was observed between the “intermediate” and “negative” groups (p=0.96, Mann-Whitney test).

Interestingly, one patient (patient #17) had no detectable SSTR-expression in his biopsy sample taken from the primary tumor in the right bronchus consistent with SSTR-PET imaging, but showed high tracer binding in all metastases.

### Prognostic value of ^68^Ga-DOTATATE-PET/CT and of SSTR2a-expression

Neither the SUV measured with PET/CT nor histological SSTR2a-expression had a prognostic value. Regression analysis (Spearman's Rho) yielded SUV_peak_ vs. PFS: ρ=0.04 with p=0.87, SUV_peak_ vs. OS: ρ=0.09 with p=0.71, T/L vs. PFS: ρ=0.13 with p=0.58, T/L vs. OS: ρ=0.17 with p=0.46, SSTR2a IRS score vs. PFS: ρ=0.17 with p=0.49, and SSTR2a IRS score vs. OS: ρ=0.05 with p=0.84.

### PRRT

In total, 5/21 patients (4/4 of the positive group and 1/6 of the intermediate group with the vast majority of lesions SSTR-positive) were considered eligible for PRRT. One patient opted for further chemotherapy, leading to a total of 4 SCLC patients undergoing 1, 1, 5 and 6 cycles of PRRT, respectively. Per cycle, 7.6±0.3 GBq of ^177^Lu-DOTATATE (patients #2, #9 and #10) / -DOTATOC (patient #17) were administered, resulting in cumulative activities of 39.6, 7.4, 7.6, and 44.2 GBq of ^177^Lu, respectively. Treatment was well tolerated by all subjects without any changes in vital signs during therapy or significant therapy-related toxicity. Especially, no kidney failure or myelodysplastic syndrome was observed.

**Figure 3:**
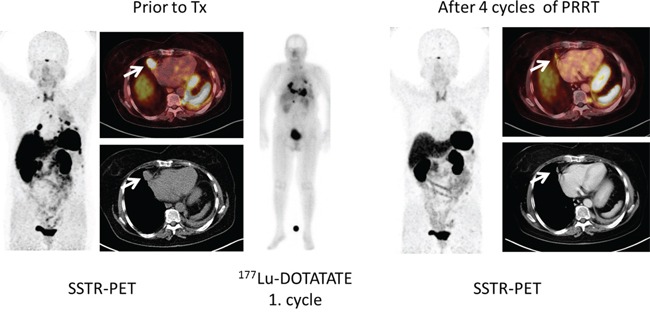
Example of SSTR-positive SCLC in a patient undergoing PRRT (patient #2) Given are maximum intensity projections as well as transaxial slices of SSTR-PET/CT in an SCLC patient (patient #2) prior to (left) and after (right) peptide receptor radionuclide therapy (PRRT). The patient had previously undergone 1^st^- (carboplatin/etoposid) and 2^nd^- (topotecan) line chemotherapy and presented with progressive disease. ^68^Ga-DOTATATE PET demonstrated intense expression of somatostatin receptors and SSTR-directed therapy was subsequently performed. After 4 cycles of PRRT, partial response could be recorded.

During follow-up, the patient undergoing 5 cycles of PRRT (patient #2) achieved partial response (Figure 3). PFS was 766 days and OS 907 days in patient #2. The patient with 6 cycles (patient #17) remained stable for 396 days with a survival of 575 days. One of the two subjects with only 1 cycle of ^177^Lu-DOTATATE reported on an improvement of his overall performance status as well as of his pain (patient #9). This patient died from pneumonia prior to his second treatment cycle. The remaining patient (patient #10) did not benefit from radiopeptide therapy and deceased 53 days after PET imaging.

## DISCUSSION

Management of SCLC remains a therapeutic challenge. Whereas cisplatin-based chemotherapeutic regimens are the mainstay of first-line therapy, a number of agents are offered for second-line treatment (usually employing the topoisomerase inhibitor topotecan). A standard third-line therapy has not been defined yet. PRRT has been investigated in this setting as an alternative for patients with high SSTR expression [9–11]. In our study, we could record a benefit from PRRT in 3 of 4 patients treated: One patient achieved partial response and another patient remained stable for more than a year. Given the median survival of <12 months in relapsed/refractory SCLC [12], both patients had a very encouraging outcome with an OS of 29 and 18 months, respectively. A third patient reported on improved performance status and pain relief after the first treatment cycle. Unfortunately, he died from pneumonia prior to the scheduled second PRRT.

Important prerequisites to SSTR-targeted therapy are a robust expression of as well as a specific binding to the target receptor. We could demonstrate a good correlation between imaging results and histopathological SSTR2a expression. However, only few patients qualified for PRRT by demonstrating higher receptor expression in tumors than in normal liver parenchyma. In our cohort, only 4 subjects presented with all SCLC lesions SSTR-positive, 6 presented with a mixture of mostly positive but also negative lesions. This finding highlights the phenomenon of tumor heterogeneity with variable features of both primary and metastatic cancer. Though a robust expression of SSTR has been described for SCLC biopsy specimens [5], receptor expression of the metastases can vary. Additionally, the dynamics of SSTR regulation under therapeutic pressure are unknown. Of note, the primary tumor biopsy sample of the patient undergoing the most cycles of PRRT was negative for SSTR expression. One might speculate that SCLC lesions with retained SSTR expression might still harbor more features of better differentiated neuroendocrine tumors and therefore be associated with a better prognosis. However, we did not observe any influence of receptor expression on progression-free or overall survival. SSTR-directed PET/CT cannot serve as a non-invasive prognostic marker. Nevertheless, it can be used as a theranostic means for PRRT assessment.

Achieved tumor doses play an important role in treatment efficacy. In our study, all results were obtained after administration of standard activities of ^177^Lu. Recent in-house dosimetry studies have suggested the feasibility of individualized PRRT with safe administration of higher activities (data not published), possibly resulting in improved outcomes. This study has various limitations. It is retrospective and comprises a very limited number of observations with only 4 patients undergoing radiopeptide therapy. Biopsies were not always obtained in close proximity to PET imaging. However, it is the first report on an anti-tumor effect of SSTR-directed PRRT. Given its excellent tolerability, it can be considered in an otherwise very limited therapeutic scenario. Future research should try to optimize both PRRT procedures (individual dosimetry prior to therapy, shorter intervals between treatment cycles) as well as focus on new potential targets. One of those targets might be represented by chemokine receptor CXCR4, which has been described to be overexpressed in a vast number of malignancies, including SCLC [13, 14]. Proof-of-concept for visualization of CXCR4 expression by a radiolabelled PET ligand [15, 16] has recently been demonstrated in SCLC patients [17]. As a compound allowing for labelling with various therapeutic α- and β-emitters is available [18], further evaluation of this theranostic approach is warranted.

In conclusion, SSTR-expression as detected by ^68^Ga-DOTATATE and/or histology is not predictive of PFS or OS in patients with advanced SCLC. However, in patients exhibiting sufficient tracer uptake, PRRT might be considered an option given its low toxicity and the absence of effective alternative treatment options. Further research on novel therapeutic targets is warranted.

## MATERIALS AND METHODS

Due to the retrospective nature of this study, our institutional review board waived the requirement for informed consent. Still, all patients gave written informed consent to receive SSTR-PET/CT imaging on a compassionate use basis for the purpose of PRRT assessment.

### Patients

Between June 2011 and October 2013, 21 patients (12 males, 9 females, age, 41-74 y) with a history of extensive stage SCLC (n=18) or LCNEC (n=3) were enrolled. All but two patients had completed 1^st^- (cis-/carboplatin/etoposide) and/or 2^nd^-line chemotherapy (topotecan, gemcitabine, ixoten) and showed progressive disease or tumor relapse on CT scans. Detailed patients' characteristics are given in Table 2.

After PET/CT, patients were monitored until death with a median follow-up of 3 months (range 0–29 months). Progression-free (PFS) and overall survival (OS) were assessed by serial CT scans (every 3 months) according to Response Evaluation Criteria in Solid Tumors (RECIST) 1.1 [19] and correlated to PET-derived parameters (SUV_peak_, tumor to liver-ratio) as well as to biopsy-derived SSTR2a and SSTR5 expression.

### SSTR-PET/CT imaging

PET/CT was performed on an integrated scanner (Siemens Biograph mCT 64, Siemens, Knoxville, USA) consisting of a Lutetium oxyorthosilicate full-ring PET and a 64-slice spiral CT. ^68^Ga-DOTATATE (114 ± 34 MBq) was injected intravenously. After a period of 40-60 min, transmission and PET emission data were acquired as previously described [20].

All imaging tests were reviewed visually by two experienced nuclear medicine physicians (C.L., R.A.W.) who were blinded to clinical data. Patients were categorized according to SSTR-positivity as “positive” (all tumor lesions PET-positive with ^68^Ga-DOTATATE uptake > liver), “intermediate” (the majority of lesions positive) or “negative” (the majority of / all tumor lesions PET-negative).

For semi-quantitative analysis, peak standardized uptake values (SUV_peak_) were calculated by assigning spherical volumes of interest of 1.5 cm diameter to the area of highest tracer uptake in tumororus tissue. In addition, a 5 cm spherical volume of interest was drawn to healthy tissue of the right lobe of the liver to determine the liver SUV_mean_, serving as reference for background activity. Tumor-to-liver ratios (T/L) were derived.

The 3 patient groups were tested by Kruskal-Wallis analysis for differences regarding the parameters age, SUV_peak_, T/L ratio, PFS, OS, and the IRS scores for the histologically assessed SSTR2a/5 expression.

### Immunhistochemical quantification of SSTR-expression

In 19/21 patients, imaging results could be compared with histologic SSTR2a and SSTR5 expression of biopsy samples from primary tumors (n=15) or metastases (n=4).

Immunohistochemistry was carried out on 10% formalin fixed paraffin embedded tissue sections according to established protocols with a routine horseradish peroxidase-based labeling technique (ADVANCE; Dako, Hamburg, Germany) and diaminobenzidine as chromogen (DAB+; Dako) and an automated platform (Freedom EVO, Tecan, Crailsheim, Germany). Polyclonal antibodies against SSTR2a (1:500, RBK 046-05, Zytomed, Berlin, Germany) and SSTR5 (1:500, RBK 051-05, Zytomed, Berlin, Germany) were used. Samples from normal pancreatic tissue were used as positive control (islet cells). Dewaxed samples were pretreated with citrate buffer pH 6.0 for 10 minutes (for SSTR2a staining) or respectively with the antigen retrieval agent TIRS-EDTA pH 9.0 for 10 minutes in a high pressure cooker (for SSTR5 staining). All immunostained sections were counterstained for 3 minutes with hematoxylin. The analysis of the stained sections was done semi-quantitatively by light-microscopy according to the immunoreactive score (IRS) by Remmele and Stegner [21]. The percentage of SSTR-positive cells was scored as follows: 0 (no positive cells), 1 (<10% positive cells), 2 (10-50% positive cells), 3 (>50-80% positive cells), 4 (>80% positive cells). Additionally, the intensity of staining was graded: 0 (no color reaction), 1 (mild reaction), 2 (moderate reaction), 3 (intense reaction). Multiplication of both scores for a given sample yields the IRS classification: 0-1 (negative), 2-3 (mild), 4-8 (moderate), 9-12 strongly positive.

### PRRT

Peptide receptor radionuclide therapy (including renal protection) was performed according to the joint IAEA, EANM and SNMMI practical guidance [22]. In brief, all patients were hospitalized for a total of 3 days. PRRT with a median of 7.6 GBq (range, 7.2-8.5) of ^177^Lu-DOTATATE/-TOC was intravenously administered over 30 minutes. Vital signs were documented during the infusion and within 7 days after administration. All patients were followed-up after PRRT including serial blood tests, kidney scintigraphy and SSTR-PET/CT. Treatment was performed with an interval of 8-12 weeks between each cycle.

### Statistical analysis

Statistical analyses were performed using IBM SPSS (version 23.0). Data were analyzed with non-parametric tests unless the Shapiro-Wilk test indicated compatibility with normal distribution. Quantitative values were expressed as mean ± standard deviation or median and range as appropriate. The tests used are reported together with the results. All statistical tests were performed two-sided and a p-value < 0.05 was considered to indicate statistical significance.

## References

[1] Govindan R, Page N, Morgensztern D, Read W, Tierney R, Vlahiotis A, Spitznagel EL, Piccirillo J (2006). Changing epidemiology of small-cell lung cancer in the United States over the last 30 years: analysis of the surveillance, epidemiologic, and end results database. Journal of clinical oncology.

[2] Cuffe S, Moua T, Summerfield R, Roberts H, Jett J, Shepherd FA (2011). Characteristics and outcomes of small cell lung cancer patients diagnosed during two lung cancer computed tomographic screening programs in heavy smokers. Journal of thoracic oncology.

[3] Wolfson AH, Bae K, Komaki R, Meyers C, Movsas B, Le Pechoux C, Werner-Wasik M, Videtic GM, Garces YI, Choy H (2011). Primary analysis of a phase II randomized trial Radiation Therapy Oncology Group (RTOG) 0212: impact of different total doses and schedules of prophylactic cranial irradiation on chronic neurotoxicity and quality of life for patients with limited-disease small-cell lung cancer. International journal of radiation oncology, biology, physics.

[4] Foster NR, Qi Y, Shi Q, Krook JE, Kugler JW, Jett JR, Molina JR, Schild SE, Adjei AA, Mandrekar SJ (2011). Tumor response and progression-free survival as potential surrogate endpoints for overall survival in extensive stage small-cell lung cancer: findings on the basis of North Central Cancer Treatment Group trials. Cancer.

[5] Tsuta K, Wistuba II, Moran CA (2012). Differential expression of somatostatin receptors 1-5 in neuroendocrine carcinoma of the lung. Pathology, research and practice.

[6] Papotti M, Bongiovanni M, Volante M, Allia E, Landolfi S, Helboe L, Schindler M, Cole SL, Bussolati G (2002). Expression of somatostatin receptor types 1-5 in 81 cases of gastrointestinal and pancreatic endocrine tumors. A correlative immunohistochemical and reverse-transcriptase polymerase chain reaction analysis. Virchows Archiv.

[7] Filice A, Fraternali A, Frasoldati A, Asti M, Grassi E, Massi L, Sollini M, Froio A, Erba PA, Versari A (2012). Radiolabeled somatostatin analogues therapy in advanced neuroendocrine tumors: a single centre experience. Journal of oncology.

[8] Imhof A, Brunner P, Marincek N, Briel M, Schindler C, Rasch H, Macke HR, Rochlitz C, Muller-Brand J, Walter MA (2011). Response, survival, and long-term toxicity after therapy with the radiolabeled somatostatin analogue [90Y-DOTA]-TOC in metastasized neuroendocrine cancers. Journal of clinical oncology.

[9] Sollini M, Farioli D, Froio A, Chella A, Asti M, Boni R, Grassi E, Roncali M, Versari A, Erba PA (2013). Brief report on the use of radiolabeled somatostatin analogs for the diagnosis and treatment of metastatic small-cell lung cancer patients. Journal of thoracic oncology.

[10] van Essen M, Krenning EP, Kooij PP, Bakker WH, Feelders RA, de Herder WW, Wolbers JG, Kwekkeboom DJ (2006). Effects of therapy with [177Lu-DOTA0, Tyr3]octreotate in patients with paraganglioma, meningioma, small cell lung carcinoma, and melanoma. Journal of nuclear medicine.

[11] Pless M, Waldherr C, Maecke H, Buitrago C, Herrmann R, Mueller-Brand J (2004). Targeted radiotherapy for small cell lung cancer using 90Yttrium-DOTATOC, an Yttrium-labelled somatostatin analogue: a pilot trial. Lung cancer.

[12] Asai N, Ohkuni Y, Kaneko N, Yamaguchi E, Kubo A (2014). Relapsed small cell lung cancer: treatment options and latest developments. Therapeutic advances in medical oncology.

[13] Kaemmerer D, Reimann C, Specht E, Wirtz RM, Sayeg M, Baum RP, Schulz S, Lupp A (2015). Differential expression and prognostic value of the chemokine receptor CXCR4 in bronchopulmonary neuroendocrine neoplasms. Oncotarget.

[14] Teicher BA (2014). Targets in small cell lung cancer. Biochemical pharmacology.

[15] Demmer O, Dijkgraaf I, Schumacher U, Marinelli L, Cosconati S, Gourni E, Wester HJ, Kessler H (2011). Design, synthesis, and functionalization of dimeric peptides targeting chemokine receptor CXCR4. Journal of medicinal chemistry.

[16] Herrmann K, Lapa C, Wester HJ, Schottelius M, Schiepers C, Eberlein U, Bluemel C, Keller U, Knop S, Kropf S, Schirbel A, Buck AK, Lassmann M (2015). Biodistribution and radiation dosimetry for the chemokine receptor CXCR4-targeting probe 68Ga-pentixafor. Journal of nuclear medicine.

[17] Lapa C, Lueckerath K, Rudelius M, Schmid JS, Schoene A, Schirbel A, Samnick S, Pelzer T, Buck AK, Kropf S, Wester HJ, Herrmann K (2016). [68Ga]Pentixafor-PET/CT for imaging of chemokine receptor 4 expression in small cell lung cancer - initial experience. Oncotarget.

[18] Herrmann K, Schottelius M, Lapa C, Osl T, Poschenrieder A, Haenscheid H, Lueckerath K, Schreder M, Bluemel C, Knott M, Keller U, Schirbel A, Samnick S, Lassmann M, Kropf S, Buck A (2015). First-in-man experience of CXCR4-directed endoradiotherapy with 177Lu- and 90Y-labelled pentixather in advanced stage multiple myeloma with extensive intra- and extramedullary disease. Journal of nuclear medicine.

[19] Eisenhauer EA, Therasse P, Bogaerts J, Schwartz LH, Sargent D, Ford R, Dancey J, Arbuck S, Gwyther S, Mooney M, Rubinstein L, Shankar L, Dodd L, Kaplan R, Lacombe D, Verweij J (2009). New response evaluation criteria in solid tumours: revised RECIST guideline (version 1.1). European journal of cancer.

[20] Buder K, Lapa C, Kreissl MC, Schirbel A, Herrmann K, Schnack A, Brocker EB, Goebeler M, Buck AK, Becker JC (2014). Somatostatin receptor expression in Merkel cell carcinoma as target for molecular imaging. BMC cancer.

[21] Kaemmerer D, Peter L, Lupp A, Schulz S, Sanger J, Baum RP, Prasad V, Hommann M (2012). Comparing of IRS and Her2 as immunohistochemical scoring schemes in gastroenteropancreatic neuroendocrine tumors. International journal of clinical and experimental pathology.

[22] Bodei L, Mueller-Brand J, Baum RP, Pavel ME, Horsch D, O'Dorisio MS, O'Dorisio TM, Howe JR, Cremonesi M, Kwekkeboom DJ, Zaknun JJ (2013). The joint IAEA, EANM, and SNMMI practical guidance on peptide receptor radionuclide therapy (PRRNT) in neuroendocrine tumours. European journal of nuclear medicine and molecular imaging.

